# Prevalence of different carbapenemase genes among carbapenem-resistant *Acinetobacter baumannii* blood isolates in Taiwan

**DOI:** 10.1186/s13756-018-0410-5

**Published:** 2018-10-11

**Authors:** Teng-Ho Wang, Yi-Shing Leu, Nai-Yu Wang, Chang-Pan Liu, Tsong-Rong Yan

**Affiliations:** 1 Divison of Infection disease, Department of Internal Medicine, Taipei City Hospital, Zhongxiao Branch, Taipei, Taiwan; 2 Graduate Institute of Bioengineering, Tatung University, Taipei, Taiwan; 3 Division of Infection Control, Taipei City Hospital, Zhongxiao Branch, Taipei, Taiwan; 4 Division of Otolaryngology, MacKay Memorial Hospital, Taipei, Taiwan; 5 Department of Medicine, MacKay Medical College, New Taipei City, Taiwan; 6 Department of Medical Research, MacKay Memorial Hospital, Taipei, Taiwan; 7 Division of Infectious Diseases, Department of Internal Medicine, MacKay Memorial Hospital, Taipei, Taiwan; 8 MacKay College of Medicine, Nursing and Management, Taipei, Taiwan; 9 Infection Control Committee, MacKay Memorial Hospital, Taipei, Taiwan

**Keywords:** *Acinetobacter calcoaceticus-Acinetobacter baumannii* (ACB) complex, Carbapenem-resistant *Acinetobacter baumannii* (CRAB), *bla*_OXA-23_-like, *bla*_OXA-24_-like, *bla*_OXA-51_-like

## Abstract

**Background:**

Although the prevalence of the carbapenem-resistant *A. baumannii* (CRAB) has increased in Taiwan, few studies have elucidated the prevalence of different carbapenemase genes in Taiwan. The first objective of this study was to identify the types and prevalence of different carbapenemase genes, and the second objective was to determine the carbapenem antimicrobial susceptibility of carbapenemase producing isolates.

**Methods:**

In total, 269 CRAB blood isolates from four medical centres in Taiwan from 1/1/2009 to 31/12/2013 were analysed. Antimicrobial susceptibilities were determined using the Vitek 2 system. Carbapenemase genes were identified by polymerase chain reaction (PCR) and sequencing. Pulsed-field gel electrophoresis (PFGE) was used to identify the different pulsotypes.

**Results:**

All 269 CRAB isolates had the *bla*_OXA-51_-like gene, while 237 (88.1%) had the *bla*_OXA-23_-like gene, and 11 (4.09%) had the *bla*_OXA-24_-like gene. Twenty-one CRAB isolates (7.81%) contained only the *bla*_OXA-51_-like gene. None of the isolates had the *bla*_OXA-58_-like gene or the metallo-β-lactamases (MBL)-encoding genes. In 28.69% of isolates with the *bla*_OXA-23_-like gene and 90.91% of isolates with the *bla*_OXA-24_-like gene, the minimum inhibitory concentrations (MICs) for imipenem were 64 mg/L or more. In 37.55% of isolates with the *bla*_OXA-23_-like gene and 100% of isolates with the *bla*_OXA-24_-like gene, meropenem MICs were 64 mg/L or more. PFGE analyses indicated that six highly similar genomes which harbored the *bla*_OXA-24_-like gene came from three different medical centres.

**Conclusion:**

Our study determined the prevalence of CRAB, the types and prevalence of carbapenemase genes, carbapenem susceptibility among CRAB isolates, and documented that the *bla*_OXA-24_-like gene had greater resistance to carbapenem than the *bla*_OXA-23_-like gene. We also demonstrated inter-hospital transmission of the highly resistant *bla*_OXA-24_-like gene.

## Background

*Acinetobacter* is a Gram-negative bacillus that is common in water and soil. *Acinetobacter* species are recognized as opportunistic pathogens of increasing relevance in healthcare-associated infections, and are particularly likely to cause opportunistic infections in intensive care units (ICUs) [1]. Analysis of healthcare-associated infection data from the Taiwan Nosocomial Infections Surveillance System (TNIS) in 2007–2012 showed that *A. baumannii* complex (AB complex) composed by *A. baumannii*, *A. nosocomialis* and *A. pittii* was among the top three common causes of infections in ICUs of medical centres and regional hospitals [2].

The *Acinetobacter calcoaceticus-Acinetobacter baumannii* (ACB) complex is composed of *A. calcoaceticus* (genospecies 1), *A. baumannii* (genospecies 2), *A. pittii* (genospecies 3) and *A. nosocomialis* (genospecies 13TU). *A. baumannii* often causes bloodstream infections, and the associated mortality rate is about 43.3% in ICUs [3]. ACB complex species have similar appearances and biochemical reactions, and cannot be distinguished by traditional biochemical reactions or by automatic or semi-automatic identification machines, such as the Vitek 2 system (BioMérieux). Therefore, ACB complex species have often mistakenly been categorized as *A. baumannii*. Precise molecular biological techniques can now be used to identify and accurately distinguish these species. *A. calcoaceticus* is less likely to cause severe disease. However, *A. baumannii*, *A. nosocomialis*, and *A. pittii* (AB complex) can cause healthcare-associated infections in ICUs. In recent years, the gradual increase of carbapenem-resistant forms of each of these species from hospital isolates has caused great concern. The resistance rate of *Acinetobacter* to carbapenems has exceeded 50% in the United States, South America, India, and China [4]. Data from the TNIS indicated the percentage of carbapenem-resistant Acinetobacter calcoaceticus-Acinetobacter baumannii (CRACB) complex increased from 49.0% in 2007 to 71.2% in 2012 among ICUs in medical centres in Taiwan, and increased from 49.8 to 63% in regional hospitals during the same period [2].

*A. baumannii* is the most common and important genospecies of *Acinetobacter* and the presence of carbapenemases is the commonest cause of resistance to carbapenems. The carbapenemases in the AB complex include carbapenem-hydrolyzing class D β-lactamases (CHDLs), which consist of OXA-51-like, OXA-23-like, OXA-24-like, and OXA-58-like genes, and class B metallo-β-lactamases, mainly VIM, IMP, and SIM [5]. Our study utilized molecular biological methods to analyze carbapenemase genes in 269 nosocomial carbapenem-resistant *A. baumannii* (CRAB) blood isolates from four medical centres in northern and central Taiwan. The first objective was to identify the types and prevalence of different carbapenemase genes, and the second objective was to determine the carbapenem antimicrobial susceptibility testing of carbapenemase producing isolates.

## Methods

### Study design

The Inclusion criteria were listed as follows: all the tested strains were isolated from infected patients from 4 tertiary medical centres in Taiwan, randomized to collect up to 100 clones of ACB complex with imipenem or meropenem MICs of at least 8 mg/L in each tertiary medical centres from January of 2009 to December of 2013. The exclusion criteria were that only the first episode was enrolled from patients with ≥2 positive blood cultures, the other blood isolates were excluded. We collected the ACB complex blood isolates using the Vitek 2 system, and further species identification of ACB complex was confirmed by analysis of the RNA polymerase β subunit (*rpo*B) gene. The prevalence of different carbapenemase genes among CRAB was performed by conventional PCR and sequencing. The imipenem and meropenem MICs were tested in all CRAB blood isolates. Additionally, tigecycline, ceftazidime, cefepime, amikacin, ampicillin/sulbactam, piperacillin/tazobactam and colistin MICs were tested for the bla_OXA-24_-like highly resistant gene only. Antimicrobial susceptibility was tested using the Vitek-2 system and interpreted according to the Clinical and Laboratory Standards Institute (CLSI) guidelines except for the MICs of tigecycline which were interpreted according to the European Committee on Antimicrobial Susceptibility Testing (EUCAST) breakpoints for *Enterobacteriaceae* spp. (MIC > 2 mg/L was defined as resistant). Pulsed-field gel electrophoresis was used to analyse the high resistant *bla*_OXA-24_-like gene pulsotypes.

Each medical centre was expected to collect 100 ACB complex blood isolates. MacKay Memorial Hospital is a 2200-bed tertiary medical centre in northern Taiwan. Taipei Veterans General Hospital is a 3000-bed tertiary medical centre in northern Taiwan. Tri-Service General Hospital is an 1800-bed tertiary medical centre in northern Taiwan. Changhua Christian Hospital is an 1500-bed tertiary medical centre in central Taiwan. These four medical centres provide both primary and tertiary care, including cancer therapy, intensive care and organ transplantation. They are premier hospitals in Taiwan.

### Collection of bacterial isolates

All the tested strains were isolated from infected patients. A total of 357 blood isolates of ACB complex, with imipenem or meropenem MICs of at least 8 mg/L, were collected from January of 2009 to December of 2013 from 4 tertiary medical centres in Taiwan. A total of 357 nonduplicate ACB complex blood isolates were collected from the 4 medical centres. ACB complex blood isolates were stored at − 70 °C in tryptic soy broth (BD, MD, USA) supplemented with 20% glycerol (*v*/v). Freezing at − 70 °C in a high percentage of glycerol were employed as cryoprotective agents. All patients were deidentified locally and re-coded. Then the blood isolates were transported to the research laboratory at MacKay Memorial hospital for further study. This study protocol (14MMHIS125) was reviewed and approved by the Institutional Review Boards of the MacKay Memorial Hospital, Taipei, Taiwan.

### Species identification

The plates were incubated under aerobic conditions at 36 °C and evaluated for growth after 24 and 48 h. Suspected colonies were further cultivated on blood agar and identified at species level using the automated Vitek MS system (bioMerieux). Antimicrobial susceptibility was tested using Vitek-2 (card AST-N196 and/or N248). All isolates were stored at − 70 °C for analysis of genetic relatedness. Genospecies identification of ACB complex was confirmed by analysis of the RNA polymerase β subunit (*rpo*B) gene according to the protocol by La Scola et al. [6].

### Antimicrobial susceptibility testing

Antimicrobial susceptibility was tested using Vitek-2 system. The imipenem and meropenem MICs were tested in all CRAB blood isolates. However, tigecycline, ceftazidime, cefepime, amikacin, ampicillin/sulbactam, piperacillin/tazobactam and colistin MICs were tested for the bla_OXA-24_-like highly resistant gene only. The MICs of tested antibiotics were interpreted according to the CLSI guidelines, but the MICs of tigecycline were interpreted according to the EUCAST breakpoints for *Enterobacteriaceae* spp. (MIC > 2 mg/L was defined as resistant). *Staphylococcus aureus* ATCC 29213, *Escherichia coli* ATCC25922, *Pseudomonas aeruginosa* ATCC 27853 were measured for quality control. All results were within quality control ranges.

### Polymerase chain reaction and sequencing

The ACB complex blood isolates were screened for the following carbapenemase genes by use of conventional PCR and sequencing: *bla*_IMP,_*bla*_VIM_, *bla*_NDM_, *bla*_SPM_, *bla*_GIM_, *bla*_SIM_, *bla*_KPC_, *bla*_GES_, *bla*_OXA-23_-like, *bla*_OXA-24_-like, *bla*_OXA-51_-like, *bla*_OXA-58_-like, *bla*_OXA-143_-like, *bla*_OXA-235_-like. The sequences of primers are shown in Table 1 [7–12]. Deionized distilled water was used as the negative control. Isolates whose sequences were previously confirmed was used as the positive control. Multiplex PCR were performed. A separate group of 2~ 10 primers (such as *bla*_*IMP*_*, bla*_*VIM*_*, bla*_*NDM*_*, bla*_*SPM*_*, bla*_*GIM*_*, bla*_*SIM*_*, bla*_*KPC*_*, bla*_*GES*_*, bla*_*OXA-23-like*_*, bla*_*OXA-24-like*_*, bla*_*OXA-51-like*_*, bla*_*OXA-58-like*_*, bla*_*OXA-143-like*_ and *bla*_*OXA-235-like*_) were used to test multiplex PCR each time. The PCR condition was one cycle of 95 °C for 10 min, followed by 40 cycles of 94 °C for 1 min, 52 °C for 1 min, 72 °C for 2 min and finally, one cycle of 72 °C for 10 min followed by cooling to 4 °C. A sample of amplified DNA was electrophoresed in an agarose gel and stained with ethidium bromide to assess the purity and size of the more multiplex PCR products. PCR products were visualized by agarose gel electrophoresis stained with ethidium bromide followed by UV light crosslinking. The PCR amplicons were purified for DNA sequencing using a ExoProStar™ reagent (GE Healthcare, UK) as per manufacturer instructions and incubated at 37 °C for 30 min, followed by incubation at 80 °C for 15 mins to inactivate the enzymes. The enzyme-treated PCR products were sequenced using the BigDye Terminator v3.1 Cycle Sequencing Kit (Applied Biosystems, Foster City, CA) following instructions provided by the manufacturer. The PCR products were purified using by gel filtration with Sephadex G-50 (GE Healthcare, UK) using spin columns in a MultiScreen™ Filtration System 96 well filter plate (Millipore) to remove unincorporated dye terminators. The final DNA sequence reading was performed in an ABI Prism 377 DNA sequencer analyzer (Applied Biosystems, Foster City, CA, USA). Sequence similarity searches were performed with the basic local alignment search tool (BLAST, http://blast.ncbi.nlm.nih.gov/Blast.cgi).

**Table 1:** Oligonucleotide primers used in this study

Primer	Sequence (5′-3′)	Reference
OXA-51-like forward	TAA TGC TTT GAT CGG CCT TG	6
OXA-51-like reverse	TGG ATT GCA CTT CAT CTT GG	
OXA-23-like forward	GAT CGG ATT GGA GAA CCA GA	6
OXA-23-like reverse	ATT TCT GAC CGC ATT TCC AT	
OXA-24-like forward	GGT TAG TTG GCC CCC TTA AA	6
OXA-24-like reverse	AGT TGA GCG AAA AGG GGA TT	
OXA-58-like forward	AAG TAT TGG GGC TTG TGC TG	6
OXA-58-like reverse	CCC CTC TGC GCT CTA CAT AC	
OXA-143-F	TGGCACTTTCAGCAGTTCCT	7
OXA-143-R	TAATCTTGAGGGGGCCAACC	
OXA-235-F	TTGTTGCCTT TACTTAGTTGC	8
OXA-235-R	CAAAATTTTAAGACGGAT CG	
Imp-F	GGA ATA GAG TGG CTT AAY TCT C	9
Imp-R	CCA AAC YAC TAS GTT ATC T	
Vim-F	GAT GGT GTT TGG TCG CAT A	9
Vim-R	CGA ATG CGC AGC ACC AG	
Sim-F	TAC AAG GGA TTC GGC ATC G	9
Sim-R	TAA TGG CCT GTT CCC ATG TG	
Gim-F	TCG ACA CAC CTT GGT CTG AA	9
Gim-R	AAC TTC CAA CTT TGC CAT GC	
Spm-F	AAA ATC TGG GTA CGC AAA CG	9
Spm-R	ACA TTA TCC GCT GGA ACA GG	
NDM-F	GGTTTGGCGATCTGGTTTTC	10
NDM-R	CGGAATGGCTCATCACGATC	
KPC-Fm	CGTCTAGTTCTGCTGTCTTG	10
KPC-Rm	CTTGTCATCCTTGTTAGGCG	
GES-C	GTTTTGCAATGTGCTCAACG	11
GES-D	TGCCATAGCAATAGGCGTAG	

### Pulsed-field gel electrophoresis

Eleven isolates with *bla*_OXA-24_-like genes among the 357 ACB complex blood isolates were typed by pulsed-field gel electrophoresis following digestion of intact genomic DNA with *Apa*I (New England Biolabs) [13]. The DNA fragments were separated on 1% (*w/v*) SeaKem Gold agarose gels in 0.5% Tris-borate-ethylene diamine tetra-acetic acid buffer using a contour-clamped homogeneous electric field (CHEF) Mapper apparatus (Bio-Rad, Hercules, CA, USA) at a potential of 6 V/cm pulsed from 5 s to 20 s for 19 h at 14 °C [13]. The gels were stained with ethidium bromide and photographed under ultraviolet light. The *Apa*I restriction profiles were initially compared by visual inspection, and isolates were considered closely related if they had differences in fewer than three bands [14]. Computer-assisted analysis using BioNumerics software (Applied Maths, Sint-Martens-Latem, Belgium) was also performed. Cluster analysis was performed by the unweighted pair group method with mathematical averaging. DNA similarity was calculated using the band-based Dice coefficient with a tolerance setting of 1.0% and an optimization setting of 1.0% for the whole profile. Isolates were considered to be in the same cluster if the similarity coefficient was greater than 87% [13].

## Results

We collected a total of 357 ACB complex isolates, including 57 from Taipei Veterans General Hospital, and 100 each from the other 3 medical centres. Among these, 334 were CRACB complex truly (93.6%); of which 269 were CRAB isolates (80.5%).

All 269 of the CRAB isolates had the *bla*_OXA-51_-like gene, a gene considered intrinsic to *A. baumannii* (Table 2) [15]. The combination of *bla*_OXA-23_-like gene and *bla*_OXA-51_-like gene had the highest prevalence, (237 isolates, 88.1%). The combination of *bla*_OXA-24_-like gene and *bla*_OXA-51_-like gene was present in 11 isolates (4.09%), and 21 isolates (7.81%) contained solely the *bla*_OXA-51_-like gene without combination with other *bla*_OXA_-like genes. None of the isolates had a *bla*_OXA-58_-like gene or any of the MBL-encoding genes.

**Table 2:** Carbapenem resistance among ACB complex blood isolates and distribution of OXA carbapenemase genes in Taiwan

Target allele(s)	No. (%) of isolates
ACB complex by Vitek 2 method	357
CRACB complex by Vitek 2 & AST method	334
CRAB by La Scola’s protocol method	269
*bla*_OXA-51_-like+*bla*_OXA-23_-like	237 (237/269, 88.10%)
*bla*_OXA-51_-like+*bla*_OXA-24_-like	11 (11/269, 4.09%)
*bla*_OXA-51_-like solely	21 (21/269, 7.81%)

Abbreviations here and below: *ACB complex Acinetobacter calcoaceticus-Acinetobacter baumannii* complex, *CRACB complex* carbapenem-resistant *Acinetobacter calcoaceticus-Acinetobacter baumannii* complex, *AST* antimicrobial susceptibility testing, *CRAB* carbapenem-resistant *Acinetobacter baumannii*

Table 3 shows the prevalence of the different carbapenemase genes among CRAB isolates from the four institutions. The highest prevalence in all 4 hospitals was for the combination of *bla*_OXA-23_-like gene and *bla*_OXA-51_-like gene (75.93–92.77%). The prevalence foe *bla*_OXA-51_-like gene solely was 2.17–22.22%, and 0–6.52% for the combination of *bla*_OXA-24_-like gene and *bla*_OXA-51_-like gene.

**Table 3:** Prevalence of CRAB/ACB complex and OXA carbapenemase genes from four hospitals in Taiwan

2009–2013	MacKay Memorial Hospital	Taipei Veterans General Hospital	Tri-Service General Hospital	Changhua Christian Hospital
CRAB/ACB complex	83/100 = 83%	54/57 = 94.74%	40/100 = 40%	92/100 = 92%
*bla*_OXA-23_-like + *bla*_OXA-51_-like	77 (77/83 = 92.77%)	41 (41/54 = 75.93%)	35 (35/40 = 87.50%)	84 (84/92 = 91.30%)
*bla*_OXA-24_-like + *bla*_OXA-51_-like	4 (4/83 = 4.82%)	1 (1/54 = 1.85%)	0 (0/40 = 0.00%)	6 (6/92 = 6.52%)
*bla*_OXA-51_-like solely	2 (2/83 = 2.41%)	12 (12/54 = 22.22%)	5 (5/40 = 12.50%)	2 (2/92 = 2.17%)

**Fig. 1:**
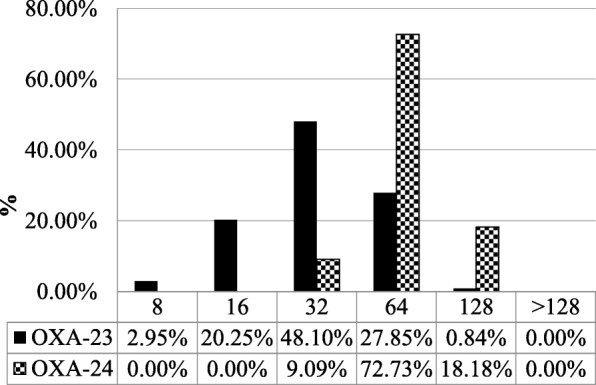
Distribution of imipenem MICs in 269 CRAB blood isolates with the *bla*_OXA-23_-like and *bla*_OXA-24_-like genes

**Fig. 2:**
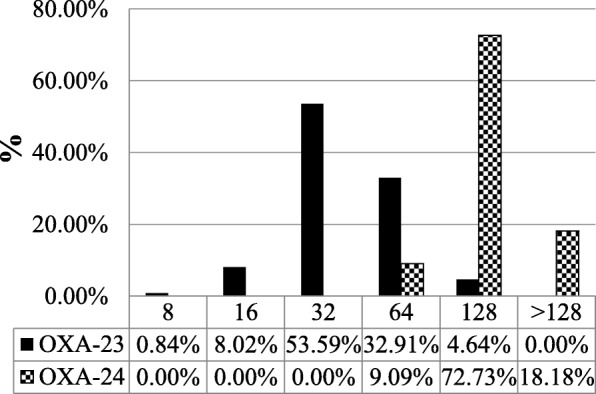
Distribution of meropenem MICs in 269 CRAB blood isolates with the *bla*_OXA-23_-like and *bla*_OXA-24_-like genes

Figures 1 and 2 show the distributions of imipenem and meropenem MICs in 269 *A. baumannii* isolates. In isolates with the *bla*_OXA-23_-like gene, 28.69% had imipenem MICs of 64 mg/L or more, and 90.91% of isolates with the *bla*_OXA-24_-like gene had imipenem MICs of 64 mg/L or more. In isolates with the *bla*_OXA-23_-like gene, 37.55% had meropenem MICs of 64 mg/L or more, and all isolates with the *bla*_OXA-24_-like gene had meropenem MICs of 64 mg/L or more. Our study showed that the *bla*_OXA-24_-like genes were more resistant than *bla*_OXA-23_-like genes to carbapenem. Hence we performed PFGE in these *bla*_OXA-24_-like highly resistant genes.

**Fig. 3:**
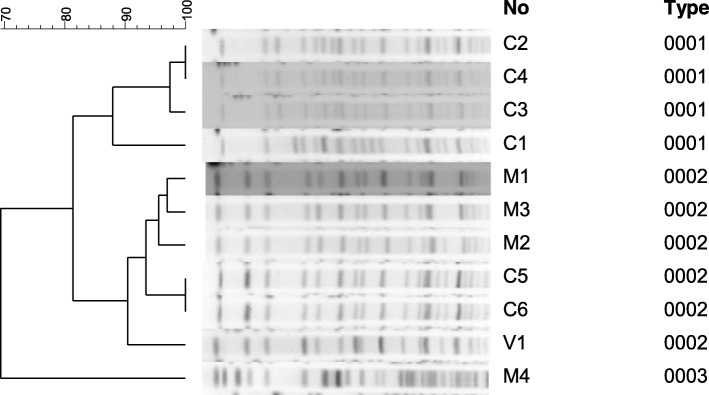
Pulsed-field gel electrophoresis results of *A. baumannii* isolates that had the *bla*_OXA-24_-like and *bla*_OXA-51_-like genes. There were 11 distinct genospecies and 3 PFGE dominant types. The scale indicates the percentage of overall genetic similarity. Isolates with the same letter were from the same hospital. M1, M2, M3, C5, C6, and V1 with the same gene cluster came from 3 different hospitals. Letter C: Changhua Christian Hospital. Letter M: MacKay Memorial Hospital. Letter V: Taipei Veterans General Hospital

PFGE analysis indicated the presence of 11 distinct strains of CRAB among isolates that had both the *bla*_OXA-24_-like gene and the *bla*_OXA-51_-like gene (Fig. 3). This analysis also indicated the presence of three main PFGE pulsotypes (Fig. 3). Interestingly, six *bla*_OXA-24_-like genes of same cluster originated from three different medical centres. It meant that inter-hospital transmission of the highly resistant *bla*_OXA-24_-like gene may occur in Taiwan. The MICs values of 11 *bla*_OXA-24_-like _+_*bla*_OXA-51_-like CRAB isolates against six antimicrobial agents are shown in Table 4. Among the eleven CRAB isolates which harbored both the *bla*_OXA-24_-like genes and the *bla*_OXA-51_-like genes, the highest sensitivity was for colistin (MICs≦1 mg/L, showed 100% sensitive). Only 9% had MICs≦1 mg/L sensitive for tigecycline, while 64% had MICs = 2 mg/L and intermediate sensitivity, and 27% MICs≧2 mg/L were resistant to tigecycline. Besides, only 9% showed sensitivity to amikacin with MICs = 8 mg/L. All eleven isolates demonstrated total resistance to cefepime (MICs ≧ 64 mg/L), ceftazidime (MICs ≧ 128 mg/L), piperacillin/tazobactam (MICs ≧ 128 mg/L) and ampicillin/sulbactam (MICs ≧ 32 mg/L).

**Table 4:** The MICs of 11 *bla*_OXA-24_-like _+_*bla*_OXA-51_-like CRAB isolates for six antimicrobial agents

OXA-24+ OXA-51 (*n* = 11)	CL		TG^a^				AMK		FEP		CAZ	PTZ	AMS		
MICs	≦0.5	1	1	2	4	≧8	8	≧128	64	≧128	≧128	≧128	32	64	128
Percentage	45%	55%	9%	64%	0%	27%	9%	91%	9%	91%	100%	100%	9%	36%	55%
Number of isolates	5	6	1	7	0	3	1	10	1	10	11	11	1	4	6
susceptibility	S	S	S	I	R	R	S	R	R	R	R	R	R	R	R

Abbreviations: *CL* colistin, *TG* tigecycline, *AMK* amikacin, *FEP* cefepime, *CAZ* ceftazidime, *PTZ* piperacillin*-*tazobactam, *AMS* ampicillin/sulbactam, *R* resistant, *S* sensitive, *I* intermediate

^a^The MICs of tigecycline were interpreted according to the European Committee on Antimicrobial Susceptibility Testing breakpoint for *Enterobacteriaceae* spp. (MIC > 2 mg/L as resistant)

The MICs of the other tested antibiotics were interpreted according to the Clinical and Laboratory Standards Institute (CLSI) guidelines

## Discussions

Antibiotic over-use has led to an increase in the prevalence of drug-resistant strains of *Acinetobacter*. In the United States, South America, India, and China, more than 50% of *Acinetobacter* species are resistant to carbapenems [4]. At the same time, carbapenem-resistant *A. baumannii* has become the most common genospecies of the CRACB complex. Our study showed that different hospitals had different CRAB/CRACB complex ratios, but a common feature was that CRAB accounted for most of our CRACB complex blood isolates. Researchers previously reported similar results for three other hospitals in Taiwan and in the US, but different results for Hong Kong, Denmark, and Norway [16–20].

In our study, all CRAB isolates had the *bla*_OXA-51_-like gene. The *bla*_OXA-23–like_ gene was the second commonest carbapenemase gene (88.10%), and its prevalence among CRAB isolates was higher than in previous reports in Taiwan. For example, Kuo et al. reported that the prevalence of the *bla*_OXA-23_-like gene was 58% in a hospital in northern Taiwan [21], while Lin et al. reported a 4.2% prevalence in a hospital in northern Taiwan [22]. Chuang et al. reported a prevalence of 7.7% in Taiwan [23]. Our data thus indicate that the prevalence of the *bla*_OXA-23like_ gene among CRAB isolates is increasing in Taiwan. In agreement with our findings, a report from Italy showed that the *bla*_OXA-23_-like enzyme was the most common carbapenemase (81.7%) [24]. Chusri et al. also reported that the prevalence of the *bla*_OXA-23_-like gene was 95% in their hospital in Thailand [25]. We think that carbapenem and/or cephalosporin antibiotics overuse could be responsible for the increased prevalence of the *bla*_OXA_-23like gene among CRAB isolates. Lack of strict infection control strategy is another cause. We also found that only 4.09% of CRAB isolates had the *bla*_OXA-24–like_ gene, similar to two other studies in Taiwan (3% and 7.7%) [21, 23]. The findings of Hu et al. report are similar to our study [26]. They found that the prevalence of the *bla*_OXA-23_-like gene was 93.5% and the *bla*_OXA-24_-like gene was 4.6% in MacKay Memorial Hospital in Taiwan. Our study differed from Hu et al. in that firstly, we investigated the prevalence of different carbapenemase genes among CRAB in multiple tertiary centres (all > 1500 beds) in northern and central Taiwan. Secondly, we compared the MICs to imipenem and meropenem between the *bla*OXA-23-like gene and the *bla*OXA-24-like gene. Lastly, we compared the MICs of the bla_OXA-24_-like highly resistant gene to ceftazidime, cefepime, amikacin, ampicillin/sulbactam, colistin and piperacillin/tazobactam. Although the *bla*_OXA-24_-like gene accounts for only a small percentage of carbapenemase genes, it confers high resistance to carbapenems (Figs. 1 and 2). Since the occurrence of the *bla*_OXA-24–like_ gene was so rare, we performed PFGE analysis and antimicrobial susceptibility tests for this group. We found that the *bla*_OXA-24–like_ gene was almost totally resistant to amikacin, cefepime, ceftazidime, piperacillin/tazobactam and ampicillin/sulbactam. In our study, the isolates with *bla*_OXA-24–like_ gene were 100% sensitive to colistin. The second recommended antibiotic is tigecycline despite the absence of Clinical and Laboratory Standards Institute (CLSI) MICs breakpoints for tigecycline.

Our study showed that the MICs to imipenem and meropenem among CRAB isolates with the *bla*_OXA-23_-like gene were much lower than that for those with the *bla*_OXA-24_-like gene. Thus, the *bla*_OXA-24_-like gene appears to confer greater resistance to carbapenem than the *bla*_OXA-23_-like gene. We also identified 3 major PFGE types of *bla*_OXA-24_-like genes in Taiwan based on ApaI digestion. Six *bla*_OXA-24_-like genes of same cluster came from 3 different hospitals (Fig. 3). This suggested that inter-hospital transmission of this resistant gene could occur in Taiwan, and may help to explain the increasing prevalence of more drug-resistant strains of CRAB in Taiwan. There are two reasons that could lead to the facilitated transmission of CRAB strains among hospitals. Firstly, in Taiwan, a patient can gain admission to any tertiary teaching hospital freely, without a doctor’s referral. Usually, the same patient had also visited several different tertiary teaching hospitals for different diseases. Secondly, after discharge, many patients carry resistant pathogens from different hospitals in which they were cared for back to the private nursing home or private respiratory care unit where they reside. The resistant strains are easily transmitted in these sites there if no strict infection control strategy. Therefore, we recommend additional infection control interventions for these patients to reduce the spread of resistant strains of CRAB.

What is the prevalence in other countries of the carbapenemase genes found in our study and do they have similar or differing antibiotic susceptibilities? What are the MICs of the *bla*_OXA-23_-like gene and the *bla*_OXA-24_-like gene to ceftazidime-avibactam, meropenem-vaborbactam or fosfomycin? Future multicenter, multi-country investigations could provide more data on the worldwide prevalence of carbapenemase genes in CRAB isolates and help the treatment of CRAB.

There were some limitations in our study. Firstly, the analysis of antibiotics consumption is not listed in our study. We could not analyse the relationship between the prevalence of the OXA genes and antibiotic drug consumption data in these 4 tertiary teaching hospitals. We were also unable to compare the relationship between the prevalence of the OXA genes and infection control strategy between these 4 tertiary teaching hospitals. Secondly, we did not perform PFGE and antimicrobial susceptibility tests on all CRAB blood isolates. Thirdly, under or over estimation of imipenem and meropenem non-sensitivity for the *bla*_OXA-24_-like gene is possible due to the small number of *bla*_OXA-24_-like genes (only 11 isolates). Large-scale studies throughout Taiwan are needed to address this limitation.

## Conclusions

Our study determined the prevalence of CRAB, the types and prevalence of carbapenemase genes, antibiotic susceptibility among CRAB isolates, and documented that the *bla*_OXA-24_-like gene showed greater resistance to carbapenem than the *bla*_OXA-23_-like gene. We also found evidence of inter-hospital transmission of the highly antimicrobial-resistant *bla*_OXA-24_-like gene in Taiwan. Results of this study are reliable because all isolates were all from blood cultures and not from bacterial colonization. Our findings are helpful in that identification of the type of gene associated with carbapenemase resistance could assist in devising strategies to reduce the transmission of CRAB strains between patients and among hospitals and thus reduce mortality from CRAB infection.

## Abbreviations

AB complex: *Acinetobacter calcoaceticus-Acinetobacter baumannii* complex
AB: *Acinetobacter baumannii*
CHDLs: Carbapenem-hydrolyzing class D β-lactamases
CHEF: Contour-clamped homogeneous electric field
CRAB complex: Carbapenem-resistant *Acinetobacter baumannii* complex
CRAB: Carbapenem-resistant *Acinetobacter baumannii*
ICUs: Intensive care units
MBL: Metallo-β-lactamases
MICs: Minimum inhibitory concentrations
PCR: Polymerase chain reaction
PFGE: Pulsed-field gel electrophoresis
TNIS: Taiwan Nosocomial Infections Surveillance System
